# Chlorine-based DUWL disinfectant leads to a different microbial composition of water derived biofilms compared to H_2_O_2_-based chemical disinfectants in vitro

**DOI:** 10.7717/peerj.9503

**Published:** 2020-07-15

**Authors:** Charifa Zemouri, Alexa M.G.A. Laheij, Catherine M.C. Volgenant, Bernd W. Brandt, Wim Crielaard, Mark J. Buijs, Egija Zaura, Johannes J. de Soet

**Affiliations:** Department of Preventive Dentistry, Academic Centre for Dentistry Amsterdam, Amsterdam, The Netherlands

**Keywords:** Biofilm, Dental unit waterlines, Disinfection, Microbiome, Sequencing, Water quality

## Abstract

**Background:**

Biofilm formation in dental unit waterlines (DUWL) may lead to health risks for dental staff and patients. Therefore, dental unit waterlines need to be disinfected, for instance by using chemical disinfectants. However, the application of chemical disinfectants may lead to the selection of specific microorganisms. Therefore, the aim of our study was to assess the microbial composition of water-derived biofilms, after a continuous exposure to maintenance doses of commercially available chemical disinfectants, in vitro.

**Methods:**

The AAA-model was used to grow water derived biofilms. The biofilms were subjected to the maintenance dose of each disinfectant. To determine the microbial composition, the V4 hypervariable region of the 16S rRNA gene was sequenced. The sequences were clustered in operational taxonomic units (OTUs).

**Results:**

The bacterial composition of biofilms in all treatment groups differed significantly (PERMANOVA *F* = 4.441, *p* = 0.001). Pairwise comparisons revealed Anoxyl treated biofilms were significantly different from all groups (*p* = 0.0001). In the Anoxyl-treated biofilms, the relative abundance of *Comamonadaceae* and *Sphingopyxis* was high compared to the Dentosept, Green and Clean and Oxygenal groups.

**Conclusion:**

We concluded that exposure to low doses of the chlorine-based chemical disinfectant Anoxyl led to a substantially different composition of water derived biofilms compared to biofilms exposed to H_2_O_2_-based chemical disinfectants.

## Introduction

Dental unit waterlines (DUWL) consist of narrow lumen tubing (Coleman et al., 2007) where low flow velocity favors microbial adhesion and biofilm formation (Walker & Marsh, 2007). Parts of the biofilm will detach and microorganisms can end up in the patient’s oral cavity, the surfaces surrounding the dental unit and in the air of the treatment room through aerosols (Coleman et al., 2007). High microbial load of effluent water, possibly containing pathogenic bacteria, poses a potential risk of infection for patients and dental staff (Coleman et al., 2007; Spagnolo et al., 2019). Besides pathogenic bacteria, also toxic bacterial products, such as endotoxins, have been found to be increased in aerosols from DULW with substantial biofilm growth, which may possess a risk for inflammation of the airways (Pankhurst et al., 2005; Szymańska & Sitkowska, 2013).

The microorganisms in the DUWL biofilms primarily originate from water, in most situations tap water. The water lines also can become contaminated by oral bacteria, from backflow when using high speed air-rotors. To prevent infection of patients and dental healthcare workers (DHCW), it is generally considered that the number of bacteria in DUWL-effluent water must meet the standard of drinking water, which is regulated nationally, and is 100 Colony forming units/mL for the Netherlands (KNMT, 2016).

Chemical disinfectants can be added to the dental unit water to reduce proliferation of biofilm microorganisms, the microbial load in effluent water and thus the risk of infection transmission. However, DUWL effluent water can still contain microbial loads above the safe water limit, as stated for the drinking water standard. This may be due to non-compliance with disinfection protocols (Volgenant & Persoon, 2018; Baudet et al., 2019; Ji et al., 2019), but also due to tolerance of the remaining biofilm to the used disinfectants. Biofilm growth is a way for microorganisms to protect themselves from antimicrobial agents, by the structure of the biofilm itself and by changing is phenotype.

So far known based on the available chemicals in the market, mainly hydrogen peroxide, silver-based chemicals or chlorine containing compounds have been used to control the microbial load in DUWLs (Abdallah & Khalil, 2011; Barbot et al., 2014; Ditommaso et al., 2016). In most studies, the disinfection effect on effluent water or planktonic bacteria was studied. It was found that low concentrations of H_2_O_2_, the most used disinfectant agent, was able to reduce the microbial concentration of planktonic cells (Orrù et al., 2010; Abdallah & Khalil, 2011; Barbot et al., 2014; Ditommaso et al., 2016). However, little is known on the effect of disinfectants on the biofilms.

The latter may result in a shift in microbial composition, depending on sensitivity or resistance to the active component of the disinfectant. Until now, only the effect of some chemical disinfectants for DUWL on specific biofilm pathogens was studied (Costa et al., 2016; Yoon & Lee, 2019). Continuous exposure of biofilms to antimicrobial agents usually results in ecological shifts in these biofilms, and just this continuous exposure during patient treatment is advised in most protocols to assure that patients and DHCW are not exposed to dangerous numbers of microorganisms. Therefore, the aim of this study was to assess the microbial composition of water-derived biofilms, after a continuous exposure to maintenance doses of commercially available chemical disinfectants, *in vitro*.

## Materials & Methods

### Biofilms and disinfection protocol

Biofilms were grown in the Amsterdam Active Attachment-model containing 24-wells plates with polyurethane discs (10 mm discs, surface area of 157 mm^2^ (both sides); ODV Rubber en Kunststoffen, Zaandam, The Netherlands) as substratum (Exterkate, Crielaard & Ten Cate, 2010). This model uses a purpose build stainless steel lid on which 24 clamps have been fixed, that hold the polyurethane discs. The clamp is placed on a 24 wells plate and inoculated with a bacterial suspension. We used an inoculation medium, generated from 20L tap water (Amsterdam, chlorine free water with less than 100 CFU/ml heterotrophic bacteria and filtered using a 0.2 µm pore-size filter system), which was stored at −80 °C in aliquots from 10^3^ heterotrophic aerobic cells until further use. The actual number of heterotrophic aerobic bacteria per well, as counted on R2A agar, was 1.6 log_10_ CFU/ml, which is similar to a normal input of a DUWL. Biofilms were grown in 10% of R2A-broth at 30 °C for 72 h. The chemical disinfectants Anoxyl (SKW Biosystems BV, end concentration 0.005% chlorine), Citrisil (Sterisil, end concentration 0.00007% silver), Dentosept (Sinrona, end concentration 0.01% H_2_O_2_), Green and Clean (GAC, Metasys, end concentration 0.02% H_2_O_2_), Oxygenal (KaVo, end concentration 0.02% H_2_O_2_) and ICX (A-dec, end concentration 0.001% H_2_O_2_ and 0.00006% silver), all commercially available in The Netherlands, were diluted in 10% R2A containing 1.6 log_10_ CFU/ml from stock, added to mimic the clinical situation, where non-sterile tap water runs into the DUWL. The chemical disinfectants were applied to the biofilms in the maintenance dose as recommended by the manufacturer and refreshed weekly for four consecutive weeks. These refreshments were made from the same bacterial stock, containing 1.6 log_10_ CFU/ml, thereby mimicking the normal use of a dental chair as close as possible.

### Biofilm sampling, DNA extraction, sequencing

Three discs were removed from the model every week and the biofilms on these discs were dispersed in 1mL sterile water by sonicating the discs for 1 min, pelleted for 15 min at 4500 rpm and stored at −80 °C for sequencing. The DNA of all samples was extracted and purified according to Cieplik et al. (Cieplik et al., 2019). In brief, the samples were added to wells of a 96-deep-well plate containing Tris-saturated phenol, 0.1 mm zirconium beads and lysis buffer and were mechanically lysed by bead-beating at 1,200 rpm for 2 min. DNA was isolated with the Mag MiniKit (LGC Genomics, Berlin, Germany).

Quantitative PCR was used to determine the bacterial DNA concentration in the biofilm samples, using universal primers specific to the bacterial 16S rRNA gene (Ciric et al., 2010). The V4 hypervariable region of the 16S rRNA gene was amplified using 1 ng DNA with 1 µM of each primer and 30 amplification cycles (Caporaso et al., 2011). Paired-end sequencing of the DNA was conducted on the MiSeq platform (Illumina, San Diego, CA, USA) with a MiSeq Reagent kit v3 and 2x251 nt at the VUmc Cancer Center Amsterdam (Amsterdam, the Netherlands). The sequence and meta data are available in the NCBI BioProject database under accession number PRJNA614901.

### Statistical analysis and data processing

The sequences of 16S rRNA gene amplicons were clustered in operational taxonomic units (OTUs) at 97% similarity as described previously (Cieplik et al., 2019). All microbiome analyses were conducted using PAST software version 3.16 (Hammer, Harper & Ryan, 2001). Statistical differences in microbial composition within the procedure was tested using Permutational Multivariate Analysis of Variance (PERMANOVA) and Principal Component Analysis (PCA). Pairwise comparisons were reported using Bonferroni corrected *p*-values. Analysis of similarity percentages (SIMPER) between samples was conducted to identify the main species that typified the microbiome. OTUs were reported at genus level. A *p*-value <0.05 was considered statistically significant.

## Results

### Microbial composition of untreated biofilms

**Figure 1:**
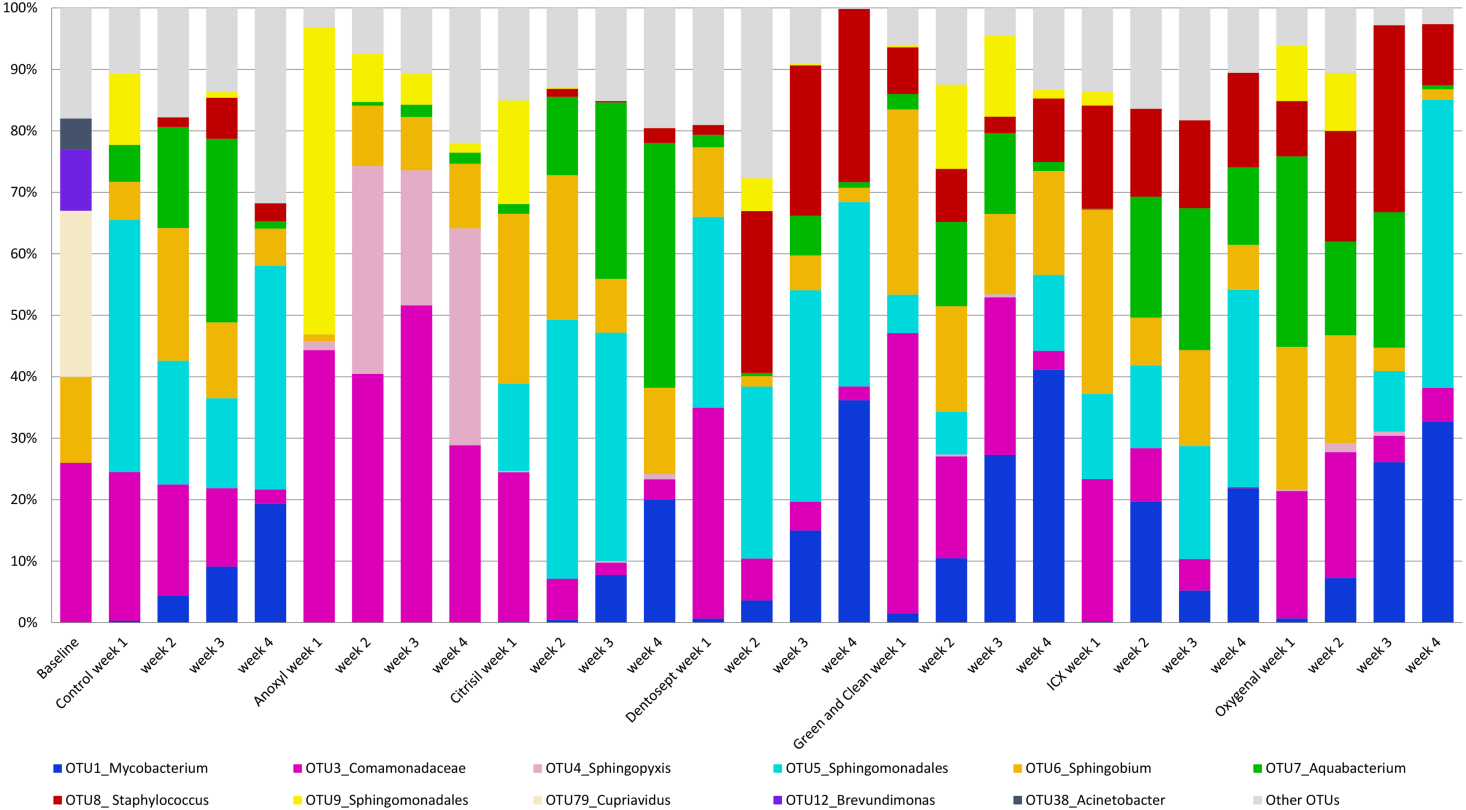
Average relative abundance of OTUs in biofilms. The average relative abundance of the ten most abundant OTUs in the biofilms, untreated (control) or challenged with different chemical disinfectants, by age of the biofilms (weeks).

The control biofilms after 72 h of growth consisted on average of 20 OTUs (range 16-27), mainly *Cupriavidus* (OTU_79, 27%), *Comamonadaceae* (OTU_3, 26%), and *Sphingobium* (OTU_6, 14%). After four weeks, the biofilms consisted mostly of *Sphingomonadales* (OTU_5, 36%), *Mycobacterium* (OTU_1, 19%) and *Comamonadaceae* (OTU_3, 6%). A full description of the relative microbiome compositions is reported in the File S1. The relative abundance of the most prominent OTU’s is presented in Fig. 1.

### Microbial abundance in biofilms exposed to chemical disinfectants

**Figure 2:**
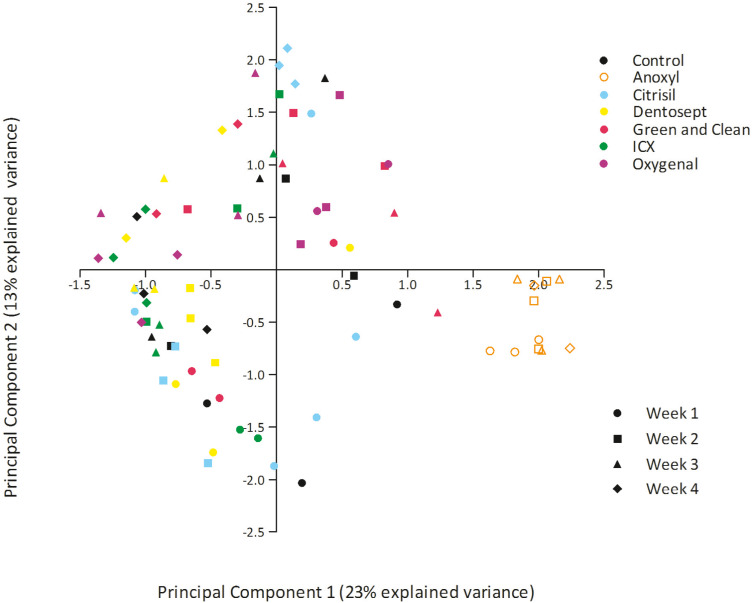
PCA plot of biofilms exposed to maintenance doses of the chemical disinfectants or left untreated (control). Each datapoint represents unique sample.

The bacterial composition of biofilms in all treatment groups differed significantly (PERMANOVA *F* = 4.441, *p* = 0.001). Pairwise comparisons revealed that Dentosept, GAC, and Oxygenal differed significantly from the control biofilm (*p* = 0.003; *p* = 0.009; *p* = 0.007). Anoxyl treated biofilms were significantly different from all groups (*p* = 0.0001). PCA analyses revealed that the samples from Anoxyl clustered separately from the rest, indicating that the bacterial community in the Anoxyl group had changed by the treatment (Fig. 2).

The microbial composition of biofilms treated by Anoxyl differed 61.9% from the control biofilm (SIMPER). Other biofilms differed between 45% and 51.7% from the control group.

PC1 explained 23% of the variance. PC1 had positive loadings for *Sphingopyxis* (OTU_4) and *Sphingomonadales* (OTU_9), while PC1 had negative loadings for *Mycobacterium* (OTU_1) and *Sphingomonadales* (OTU_5). In the Anoxyl-treated biofilms, the relative abundance of *Comamonadaceae* (OTU_3) and *Sphingopyxis* (OTU_4) was high compared to the Dentosept, GAC and Oxygenal groups. The proportion of *Mycobacterium* (OTU_1) increased in the Dentosept, GAC and Oxygenal groups over time, while it was absent in the Anoxyl-treated biofilms (Fig. 1). The same trend for *Mycobacterium* (OTU_1), though less pronounced, was observed in the control biofilms.

Anoxyl-challenged biofilms were dominated by *Comamonadaceae* and *Sphingopyxis* and lacked genus *Mycobacterium*. H_2_O_2_-challenged biofilms were most similar to the control biofilms, where *Mycobacterium* and *Sphingomonadales* species were more abundant.

## Discussion

The presented study showed the microbial composition of the treated and untreated water derived biofilm over 4-week time period. The biofilms were dominated by *Mycobacterium, Sphingomonadales, Sphingopyxis*, and *Comamonadaceae*. The microbial composition between the untreated biofilms and those exposed to biocides containing H_2_O_2_ did not differ. The latter might also be due to the small sample number. Exposure to chlorine-based biocide (Anoxyl) led to a substantially different composition of the biofilm compared other treatment groups. Untreated biofilms and those exposed to H_2_O_2_ were dominated by *Mycobacterium* and *Sphingomonadales*. Biofilms exposed to chlorine consisted mainly of *Comamonadaceae* and *Sphingopyxis*. The presented results show that the relative abundance of *Mycobacterium* increased weekly in the H_2_O_2_-group. This time factor showed that continuous exposure to biocides may change biofilm composition and it can be questioned what will happen when these biofilms are treated with H_2_O_2_ for many years. *Mycobacterium* was found to be resistant in water treatment to H_2_O_2_ (Yang et al., 2017). If *Mycobacterium* was exposed to chlorine treatment, the genus was fully absent in all taken samples which is in line with treatment results in waste water studies (Norton & LeChevallier, 2000; Oriani, Sierra & Baldini, 2018). Furthermore, a chlorine-based DUWL disinfectant proved to be successful in reducing the number of aerobic bacterial counts in effluent water (O’Donnell et al., 2009). *Mycobacterium* has been previously isolated from DUWL and can be retrieved from aerosol samples (O’Donnell et al., 2011; Walker et al., 2004; Castellano Realpe et al., 2020). The presence of this species is of a public health interest since it can cause infections in immunocompromised patients (Koh, 2017).

The absence of sample clustering in the PCA-plot might be due to a low diversity of the baseline biofilms which could be a result of the low number of microorganisms in the inoculum. Biofilms are unique ecosystems which microbial composition depends on the inoculum size, inoculum composition and inoculum diversity in combination to the exposure to biocides. Differences in microbial composition endorse the argument that biofilms in a particular DUWL, even when treated with the same disinfectant agent, are unique and may differ from other similar units in the same clinic (Abdallah & Khalil, 2011). A high relative abundance of a single species decreases the diversity which might lead to a resistant biofilm. One study on microbial shifts in waste water treatment suggests that a combination of different biocides may be the most powerful strategy for treatment efficiency (Yang et al., 2017).

## Conclusions

The study of changes in biofilm microbiome composition is important for improved infection risk assessment and infection control strategies targeting the DUWL. Prolonged application of the same chemical disinfectant to DUWL may lead to tolerance and selection of the bacteria in the remaining biofilm. This may result in thicker biofilms and a potential risk for patients and staff. Moreover, thick biofilms tend to be more resistant against biocides, which also may affect the biofilm composition (Bridier et al., 2011). Biofilms with a dominance of a single species might be aerosolized through dental instruments and cause pulmonary infections as in case of *Mycobacterium*

##  Supplemental Information

10.7717/peerj.9503/supp-1 File S1 OTU table of sequenced biofilms The total OTUs of each biofilm sample and the genus names determined in column CL.

## Additional Information and Declarations

### Data Availability

The following information was supplied regarding data availability: Data is available at NCBI: PRJNA614901.
